# On the Popular Study of Metaphysics

**Published:** 1853-04-01

**Authors:** 


### Art. V.—ON THE POPULAR STUDY OF METAPHYSICS.

The study of mental philosophy in all its branches has received an untoward impulse. It promises to become fashionable. We now seldom take up "The Publishers' Circular" without observing the announcement of some new work on logic, psychology, or metaphysics. Here we find advertised in startling emblazonry, "Logic for the Million;"* there, streaming from the north, "An Introduction" to the "Philosophy of the Mind, intended especially for the use of students in the Universities;"† and last, not least, we have lying before us a "re-opening lecture" on the "Metaphysics of Education," which was

* "Logic for the Million; or, The Art of Reasoning." By a Fellow of the Royal Society. 12mo. London.

† "An Introduction to Mental Philosophy, in Two Parts, intended especially for the Use of Students in Universities." By George Ramsay, B.M. Edinburgh, 1853.

delivered on "the first day of term,"—for scholars, like lawyers, keep "terms" at school,—at St. Mary's Hall, St. Mary's Road, Canonbury,—an institution for female education.* What metaphysics can practically have to do with the education of young ladies, or the education of young ladies theoretically with metaphysics, might appear a problem; but unless a schoolmistress be cognizant of the existence of certain mental faculties, how can she, it may be argued, philosophically rouse those of her scholars into activity? How, without gauging their capacities, can she apportion to each the lesson or lessons which they may be qualified to learn—whether in needlework, geography, tambour work, or history? Without knowing what philosophers have said about "consciousness," "attention," "conception," "abstraction;" the "association of ideas"—"memory," "imagination," and "judgment," how can she possibly preside successfully over the development of those mental faculties, which it is her duty and business to direct and control? Furthermore, without knowing something of the laws of memory, how can she teach her pupils to remember the declensions of nouns, adjectives, and verbs, in the English, French, or German grammar? to say nothing of syntax, prosody, or the composition of themes? Without exciting and judiciously training the imagination and judgment, how can she succeed in teaching any young lady to excel in playing the piano-forte or harp, or in executing graceful problems of any kind, even in crochet-work? Then there is a state of "negative consciousness," (well described by our metaphysician, at page 7, in the lecture before us,) to which young ladies are particularly liable at school, viz., a state of self forgetfulness (*query* idleness,) in which they "lose themselves, philosophically speaking, in the subject and object," and which requires considerable address on the part of the teacher to cure on sound metaphysical principles. Furthermore, as Locke has clearly shown, (at least to the satisfaction of his disciples,) all human knowledge, including needlework, must be acquired either by sensation or reflection. So that schoolmistresses can have little chance of prospering who know nothing about the sensations and reflections of the pupils committed to their charge. A preliminary lecture, therefore, on the "Metaphysics of Education" was happily enough chosen to inaugurate the re opening of the Ladies' School, St. Mary's Hall, Canonbury!

Some testy and querulous bachelor-philosophers may however pretend to argue, that ladies, even schoolmistresses, governesses, and their assistants, have no business with metaphysics, and that such speculations do not come legitimately within their sphere; to which we would fain

* St. Mary's Hall, St. Mary's-road, Canonbury: an Institution for Female Education. The Re-opening Lecture on Metaphysics of Education. By the Rev. J. F. Denham. London: Spiller, 1853.

retort, that the intellectual faculties of some ladies may be as capable of expansion and exaltation as those of the sterner sex. Witness the fact attested by Descartes himself, that Christina, Queen of Sweden, and Elizabeth, daughter of Frederick, King of Bohemia, the mother of our Royal family, thoroughly understood the abstruse principles of his philosophy. Nay, Descartes declares, that the Princess, though very young when he wrote his Principia, was the only person he knew who thoroughly understood not only his philosophical, but even his most profound mathematical disquisitions. Who then shall impeach the intellectual capacities of the fair sex? Did not the celebrated philosopher Euler, when he addressed those charming letters on the most subtile problems in philosophy to a German Princess, fully recognise her ability to comprehend the most recondite truths? and, do we not find Madame de Stael criticising, even to the admiration of Dugald Stewart, the philosophy of Hobbes, Kant, Leibnitz, Wolf, Fichte, which she appears to have thoroughly mastered? Who will dispute the erudition of Madame Darcier? Who gainsay the profound knowledge of Mrs. Somerville, not only in astronomy, but in all the physical sciences? "Woman has yet," says an accomplished writer, "to learn her nature and its powers. Man has yet to learn what woman is, and to what she is destined. It is rather in capabilities than in existences that humanity must be studied. . . . . . Before woman can advance, man must love her for the higher, not the lower faculties of her being."* There is much truth in the remark, nor will the divine mission of woman be contravened by her being capable of appreciating the knowledge to which some men dedicate their lives.

But metaphysics—alas! metaphysics—those difficult, we might almost say sacred, problems, which were formerly conceived to be fit subjects only for the investigation of philosophers, are now in the hands of the multitude. The secrets of initiation are no longer necessary; they have been scattered to the winds; the holy of holies has been ransacked; and while we yet write we hear the inarticulate murmurs of the crowd without threatening the invasion of the spiritual world. But in the midst of all this popular ferment, and bustle, and public lecturing, are we really making any advancement? Are we over-stepping the boundary line which appears to have circumscribed so inexorably the knowledge of the ancient philosophers? A rocking-horse,—a child's rocking-horse,—shall be impetuously moved backwards and forwards, yet will it not advance a jot. Here we have a type of movement without progress, closely resembling some of those hobby-horses, which our metaphysical

* "The Ministry of the Beautiful." By Henry James Slack. London: Bentley. 1850. pp. 63—64. A very charming little work, but which, we fear, is not so well known as it deserves to be.

philosophers are wont to ride. Why should not such a rocking-horse be set before the threshold of every ladies'-school? Outward signs beget inward confidence; and it is now-a-day the fashion to teach the gentler sex every branch of literature and science. "Schools have multiplied," says Mr. De Quincey,* and measures are in progress for bringing education, like water and gas, almost to every cottage door." True! But he adds significantly,—"the external machinery" of education has hitherto achieved little,—"the tubes for distributing the water are better far than once they were; but the waters themselves that must slake our thirst are as turbid as ever." The water is impure, the gas noxious; what then have we gained by overthrowing the ancient Temples of philosophy, and macadamizing with their ruins the highways and byways of modern literature? Assuredly nothing! We advance not a single step by re-echoing doctrines and opinions which were the subject-matters of controversy between Hobbes and Wallis,—Locke and Stillingfleet,—Hume and Reid. Why fix our gaze on so unprofitable a retrospect, and retrace our steps only to return along the same dull, dreary, weather-beaten road? We must not trumpet forth our own praises and talk loudly of the progress of intellectual philosophy, and yet keep moving within the same circle; enunciating principles older than the foundation stones of our own universities; or keep our eyes fixed on the same horizon, and perform the same gyrations; turning round and round, as Goethe truly remarked, like kittens playing with their own tails.

According to Mr. Samuel Warren, the progress we are making in mental philosophy is more apparent than real; there are few, he says, bold original thinkers among us who, like Descartes and Kant, look beyond the immediate precincts of the age. In philosophy, as in the starry heavens, there are, nevertheless, what Humboldt describes as "gaps," which only some far-seeing eye has the power to perceive and penetrate. Yet have we lectures, popular lectures too, upon metaphysics, delivered at royal institutions and ladies' schools. But, be it observed, that when Sydney Smith delivered his lectures on moral philosophy to the *élite* of fashion in Albemarle Street, he was deeply impressed with the gravity of his subject. The very word "metaphysics" he handled with as much caution as if it had been a hand-grenade.

"There is a word," he exclaimed with his deep, sonorous, warning voice, "of dire sound and horrible import, which I would fain have kept concealed if I possibly could; but as this is not feasible I shall even meet the danger at once, and get out of it as well as I can. The word

* Article on Logic in "Hogg's Instructor," 1852. When will this accomplished scholar favour us with an edition of his own collected writings? His articles in Blackwood would alone be a valuable contribution to modern literature!

to which I allude is that very tremendous one of 'metaphysics,' which in a lecture on moral philosophy seems likely to produce as much alarm as the cry of 'fire' in a crowded play house; when Belvidera is left to cry by herself, and every one saves himself in the best manner he can. I must beg my audience, however, to sit quiet; and, in the meantime, to make use of the language which the manager would probably adopt on such an occasion: I can assure ladies and gentlemen there is not the smallest degree of danger."*

Neither was there the least cause for any real alarm when the Rev. J. F. Denham addressed the ladies at St. Mary's Hall, Canonbury; albeit some of the said pupils who might have been present may, intuitively, have apprehended some danger from the idea of a metaphysical task "looming in the distance;" but we can vouch for it, (having had the pleasure of being there,) that the lecturer delivered himself of his budget in a cool, fluent, and graceful manner, which won the confidence and admiration of his audience. Nevertheless, there was considerable temerity, if not recklessness, in the way he used, as appears to us even now, on the printed wrapper of the lecture, the word "metaphysics," which may have reminded some of the younger listeners of the line in one of Watts's favourite hymns—"There's none but a madman would throw about fire," &c., and "Say it was only in sport."

This ominous and mysterious word, "metaphysics," has been used in a very vague and indeterminate manner by many philosophers, symbolizing, aptly enough, the shadowy and evanescent character of some of their most favourite theories. By the ancients it was clearly enough applied to all those abstract *à priori* speculations which did not come tangibly within the sphere of physical science. The attributes of the Deity, the origin of the world, the occult qualities of matter, the laws of motion, the nature of angels and demons, the essence of the soul, or mind, came within the comprehensive range of metaphysics, as did also the whimsical conundrums which puzzled the wits of the schoolmen in the middle ages: such as whether an angel could traverse from one point of the heavens to another without passing over the intermediate cerulean space; whether two lines running parallel would ever meet at either end; and other as recondite problems which Zadkiel himself might even now be puzzled to resolve. There is really, however, no mystery connected with the history or proper application of this word. "In what manner," says Dugald Stewart, "the philosophy of the human mind came to be considered as a branch of metaphysics, and to be classed with the frivolous sciences which are commonly included under the same name, is well known to all conversant with literary history. It may be proper, however, to

* "Elementary Sketches of Moral Philosophy." By the Rev. Sydney Smith. London. 1850. Chap. I., p. 3.

mention here, for the information of some of our readers, that the word metaphysics is of no older date than the publication of Aristotle's works by Andronicus of Rhodes, one of the learned men into whose hands the manuscripts of that philosopher fell after they were brought by Sylla from Athens to Rome. To fourteen books in these manuscripts which had no distinguishing title, Andronicus is said to have prefixed the words τα μετα τα φυσικα (the books after Physics) either to denote the place which they occupied in Aristotle's own arrangement immediately after the Physics, or to point out that which it appeared to the editor they ought to hold in the order of study." "Notwithstanding the miscellaneous nature of these books" (continues Dugald Stewart) "the Peripatetics seem to have considered them as all belonging to one science, the great object of which they conceived to be first to treat of those attributes which are common to matter and to mind; secondly, of things separated from matter, particularly of God and of subordinate minds which they supposed to carry on the physical change exhibited in the universe. A notion of metaphysics, nearly the same, was adopted by the Peripatetics of the Christian church. They distinguished its two branches by the titles of Ontology and Natural Theology; the former relating to being in general, the latter to God and to angels. To these branches the schoolmen added the philosophy of the human mind, as relating to an immaterial substance, distinguishing this last science by the title Pneumatology." We thus trace the origin of the word pneumatology to the scholastic ages; and it is not unimportant to observe that the ancients, to designate the soul or spirit, always selected the most subtile, evanescent, and aeriform object they could conceive. The life, the animus, symbolised the soul; the πνευμα, or breath, conjoined with the word λογος, a discourse upon the mind. In the sixteenth century the more appropriate title of Psychology was introduced. We are informed by Blakey that the first writer who applied this word to intellectual studies was Godenius, in a work entitled, *Psychologia: hoc est, de hominis perfectione animæ*, published at Marbourg in 1590.* Notwithstanding Dugald Stewart has spoken disparagingly of the term,† it was highly approved of by Dr. Campbell and Dr. Beattie, and has recently been stamped with the authority of Sir William Hamilton, whom Cousin has designated "le plus grand penseur et le plus grand critique de notre siècle."‡ "The word Pneumatology," says Sir William, "is now properly superseded by the term Psychology, to which no

* "History of the Philosophy of the Human Mind." By Robert Blakey. Vol. ii. p. 138.

† See Note, "Elements," Op. cit. Vol. i. p. 19.

‡ "Fragments Philosophiques." Avertissement, tome i., p. 3.

competent objection can be made, and which affords us what the various clumsy periphrases do not, a convenient adjective—Psychological."*

We are, therefore, at a loss to understand why Mr. Ramsay, in his "Introduction to the Study of Mental Philosophy," should recur to the old indefinite word, Metaphysics,† to signify, as he explains it, "pure mental science."‡ Neither the etymology nor the history of the word justifies such an interpretation; and our excellent lecturer, Mr. Denham, falls into the same error. The lecture before us is not upon metaphysics, in the peripatetic signification of the word, but upon psychology; and we are therefore surprised to find ourselves carried a century or more back by the declaration, "our topic, Metaphysics of Education, embraces that department of metaphysics which consists of mental philosophy." In challenging the correctness and propriety of this language, we must not be accused of evincing a disposition to split straws about trifles, or of fencing with mere shadows, for accuracy of language in all sciences, more especially in those which are founded upon *à priori* reasoning, is of vast importance. "In words contemplated singly," says a recent and accomplished author, "there are boundless stores of moral and historic truth. . . . . Words often effectually embody facts of history. . . . . The language of a people presents the marks and vestiges of great revolutions."§ If this be true as affects our social history, how much more strikingly do words, bare scientific words, embodying the meaning and object of a science, indicate our intellectual progress. Metaphysics, pneumatology, psychology, are terms which stand, century after century, in advance of each other, like land marks, indicating successive epochs in mental philosophy. The "Metaphysics of Education" adapted to a lady's school, would certainly, looking to the peripatetic history of the word, have suggested to us some recondite speculations respecting the properties and qualities of slates and rulers, work-bags and tambour-boards. We should have anticipated a luminous disquisition upon the English and French systems of numeration and notation, which would enable the intelligent pupil to calculate with ease and certainty any combination of numbers, from units to millions, and from millions to quintillions or sextillions. We should have looked for a philosophical explication of the theory of

* "Reid's Collected Writings." By Sir William Hamilton, p. 218.

† The very vague and loose manner in which the word metaphysics has been used by some philosophers has rendered the word, with the vulgar, synonymous with the unintelligible. Hence the blacksmith of Glamis' description of metaphysics in good broad Scotch:—"*Twa fouk disputin thagither; he that's listenin disna ken what he that's speakin means, and he that's speakin disna ken what he means himsel—that's metaphysics!*"

‡ Ramsay, Op. cit., p. 113.

§ "The Study of Words. Lectures addressed originally to the Pupils at the Diocesan School, Winchester." By Richard Chenevix Trench. London: Parker, 1853.

music, the mystical relation between crotchets and quavers, the principles of harmony and modulation, the structure of the diatonic scale, and the abstract nature of conjunct tetrachords : but instead of all this the lecturer has attempted only to propound to us, in ‘ a didactic and cursory" manner, the constitution of the mind; and in order, we presume, to facilitate the task of analysis, he has set out with lopping off with one fell swoop those "distinct powers of the mind," which philosophers, reflecting upon their own consciousness, have taken so much pains to classify. In other words, we cannot help feeling that our lecturer has been picking our brains of our intellectual faculties, which upon such an occasion, and in the presence of so many ladies, we must say was hardly fair. To explain, however, so bold an attempt it may be necessary to state, that when Dr. Thomas Brown, one of the most ingenious and distinguished philosophers of the Scottish school, seceded from the doctrines of his preceptors, Reid and Dugald Stewart, he attempted to establish a new system of philosophy, in devising which he adopted a peculiar phraseology, suggested a new classification of mental phenomena, and proposed to abolish any distinctive recognition of the powers of mind, apart from the states in which the mind itself exists. This very convenient notion our lecturer sets out with adopting; hence, after quoting from Brown the assurance, that "the mind is not a mere bundle of faculties, but a unity capable of passing into consecutive states," he has the satisfaction of announcing that it is "unimportant whether we retain the old classification of the faculties or adopt a new one." This of course opens up a *viâ regia* in mental philosophy, which apparently facilitates very greatly the progress of the student. The moment we reflect upon this assumption we shall find that this renunciation of the powers or faculties of the mind is a deception, for the mind must have the intuitive power, or faculty, of passing into those very states which Brown has himself described. Using the word faculty in the restricted and proper sense contended for by Locke,* it is clear that the power of performing any operation, physical or mental, must be ascribed to the power which enables the agent to perform different acts, and enter into different states. To what then does Brown's objection amount? Mr. Denham must be well aware that there is one flesh of fowl and another of fish. The soaring faculty is not the same as the diving faculty; neither is that faculty which gives wings to the imagination of the poet identical with that faculty which enables the mathematician to propound a problem sunk "ninety and nine fathom deep" in mystery. It is true, that Brown had reason to complain in his time (as also had Locke) that the word faculty was by many authors greatly abused.

* "Essay concerning the Human Understanding." Book ii. c. 21, sec. 17.

"No sooner," he observes, "were certain affections of the mind classed together as belonging to the will, and certain others as belonging to the understanding,—that is to say, no sooner was the mind existing in certain states denominated the understanding, and in certain other states denominated the will, than the understanding and the will ceased to be considered as the same individual substance, and became immediately as it were two opposite and contending powers in the empire of mind, as distinct as any two sovereigns with their separate nations under their control; and it became an object of as fierce contention to determine whether certain affections of the mind belonged to the understanding or to the will, as in the management of political affairs, to determine whether a disputed province belonged to one potentate or to another. Every new division of the faculties of the mind indeed converted each faculty into a little independent mind, as if the original mind were like that wonderful animal, of which naturalists tell us, that may be cut into almost an infinite number of pieces, each of which becomes a polypus as perfect as that from which it was separated. The only difference is, that those who make us acquainted with this wonderful property of the polypus, acknowledge the divisibility of the parent animal, while those who assert the spiritual multiplicity are at the same time assertors of the absolute indivisibility of that which they divide."*

There is here some little misapprehension. The philosophers who recognise the existence of different faculties or powers of the mind, do not imagine that the mind is "a mere bundle of faculties" (page 7, Lecture), but they fully admit, from the evidence of their own consciousness, that the mind is always in itself an "unity capable of passing into consecutive states;" the rapidity of which transition is so marvellous as to escape the observation of the thinker himself. Hence the following very striking illustration :

"When Captain Head was travelling across the Pampas of South America, his guide one day suddenly stopped him, and pointing high into the air, cried out : 'A lion !' Surprised at such an exclamation, accompanied with such an act, he turned up his eyes, and with difficulty perceived, at an immeasurable height, a flight of condors soaring in circles in a particular spot. Beneath this spot, far out of sight of himself or guide, lay the carcass of a horse, and over that carcass stood, as the guide well knew, a lion, whom the condors were eyeing with envy from their airy height. The signal of the birds was to him what the sight of the lion alone would have been to the traveller, a full assurance of its existence."

"The sight of the condors" (says the excellent logician from whom we have quoted the above passage) "convinced him that there was some carcass or other; but, as they kept wheeling far above it, instead of swooping down to their feast, he guessed that some beast had anticipated

* Dr. Brown, "Philosophy of the Mind." Lecture xvi.

them. Was it a dog, or a jackal? No! The condors would not fear to drive away or share with either. It must be some large beast; and as lions abounded, or had been seen in the neighbourhood, he concluded that one was here. These steps of thought, at least, and probably many more, rushed through his mind with the proverbial swiftness of thought, but they were summed up in the words—"a lion!"*

We have here a very striking, and indeed beautiful example of the rapidity with which an indefinite series of mental processes may be consecutively carried on. "Daily and hourly" (continues this admirable author), "we run through similar or more complicated trains of thinking, with no more consciousness of the several links than the organ-player has of each note he strikes in a rapid passage of full harmony. Yet have we, in the instance of the American guide journeying over the Pampas, evidence of the activity of those faculties which he was in the habit doubtless of exercising in pursuing his vocation. We observe the instantaneous operation of the faculties of perception, comparison, association of ideas, and memory, leading to a direct and palpable generalization. The inference of the guide was as correct, without knowing the successive steps of his reasoning, as if he had elaborated it by the rules of logic, and arrived at the conviction through the terms and propositions which constitute a syllogism." All this, too, was accomplished with inconceivable quickness, outspeeding, in our conception, the swiftness of the lightning, 'which doth cease to be, ere one can say it lightens!'

Now, in studying psychology, the philosopher who would seek to analyze the operations of the mind, must set out with observing and classifying its several powers and faculties; not that these powers and faculties are in themselves anything separate or distinct from the mind itself; but inherent in its constitution, just as the laws of gravitation are inherent in matter. We cannot, indeed, conceive the existence of matter independent of the laws which govern its conditions; neither can we imagine the existence of mind unendowed with those powers and faculties, by virtue of which it is enabled to pass transitionally into its different and consecutive states; we say transitionally, because the stream of thought is ever continuous—it is never interrupted—the unity of consciousness never disturbed. We advance not a step by denying the expediency of recognising and classifying the faculties with which the mind is so manifestly endowed; and we concur with Mr. Morell in contending "strongly against giving up the use of the words 'power,' 'faculty,' and other similar expres-

* "Outline of the Laws of Thought: a Treatise on Pure and Applied Logic." By William Thomson, p. 74.

sions, which keep constantly before our view the native activity or spontaneity of the human mind, for the purpose of substituting in their place the phraseology which represents all mental phenomena as states produced by extraneous causes."*

The pupils disposed to study psychology at St. Mary's Hall, Canonbury, cannot, we are convinced, adopt any better vocabulary than the one which Locke, Reid, and Dugald Stewart were content to use. So expressive, indeed—nay, so indispensable is the word faculty to indicate the powers of the mind, that Brown himself repeatedly uses it; and one worthy lecturer, after throwing his "bundle" over his shoulder, gathers up its fragments, and arranges a classification of them, which is not only exceedingly imperfect, but, in some points over-redundant, inasmuch as he presses conditions of mind into the service of the faculties, which ought not to be so enumerated. We hear nothing of the rudimentary faculty of perception, but we are informed that "the first faculty, or mental state, both in the natural and philosophical order of the subject, is consciousness." We marvel that our author, having declared himself a disciple of Brown's, should have overlooked his preceptor's chapter upon this subject, as well as the very luminous reasoning of Sir William Hamilton, who has demonstrated, as far as the force of argument can go, that consciousness is the universal condition of all intelligence, underlying and sustaining every mental act, operation, state, mode, modification, or by whatever other name any mental phenomena may be designated. "Consciousness (observes Sir William) is not one of the special modes into which our mental activity may be resolved; but the fundamental form—the generic condition of them all. Every intelligent act is thus a modified consciousness; and consciousness a comprehensive term for the complements of our cognitive energies."† Arguments of a similar character may be urged a ainst Dugald Stewart, for having enumerated attention among the number of the faculties.‡ "If attention (exclaims Sydney Smith) be a faculty, it is certainly *a discovery*, for nobody had ever so classed it before Mr. Stewart."§ And our critical philosopher then adds—"Which is the most important consideration for the governesses of St. Mary's Hall, Canonbury, that 'whether it be so or not, is of no consequence in practice, for nobody has ever been ignorant of the importance and efficacy of attention, whether it

* Morell, "Historical and Critical View of the Speculative Philosophy of Europe," vol. ii. p. 26.

† Sir W. Hamilton's "Discussions on Philosophy and Literature," p. 47. See also an excellent article on Speculative Philosophy in the *British Quarterly Review* for November, 1852.

‡ "Elements of the Philosophy of the Human Mind," vol. i. chap. ii.

§ "Lectures on Moral Philosophy," Op. cit., p. 14.

be one thing or another thing.'" No public orator, or barrister, ever summed up an argument more felicitously than Sydney Smith; he always applied the axe to the root of the tree; and with a good practical aim brought the *cui bono* clearly into view. We would like to have heard him descant on the metaphysics (as Mr. Denham terms it) of ladies' education. But we are now exceeding our limits; and although the dinner bell may not yet have rung in St. Mary's Hall, we will, before concluding our observations, confess that we see no reason against a metaphysical gymnasium being attached to every ladies' seminary in the kingdom. But we would recommend Mr. Denham during the next midsummer vacation to revise many of his views. We hail, however, his present lecture as an indication that mental philosophy is, as we stated in the commencement of the article, exciting public interest; and in assuming a popular form, many discrepancies and errors may naturally be expected. When man becomes, in the language of Plato, "a hunter after truth," he then enters nobly upon his spiritual career. He has a reason, too, for the faith that is within him; because he is actuated by the intuitive conviction, that the knowledge he seeks will be eventually revealed to him; and with this sentiment profoundly impressed upon us, we annex the following admirable passages from Sir William Hamilton's "Essay on the Philosophy of Perception:"

"Did the Almighty (says Lessing), holding in his right hand '*truth*,' and in his left, '*search after truth*,' deign to proffer me the one I might prefer,—in all humility, and without hesitation, I should request '*search after truth*.' We exist only as we *energize;* pleasure is the reflex of unimpeded energy; energy is the *mean* by which our faculties are developed; and a higher energy the end which their development proposes. In *action* is thus contained the existence, happiness, improvement, and perfection of our being; and knowledge is only precious as it may afford a stimulus to the exercise of our powers, and the condition of their more complete activity. Speculative truth is, therefore, subordinate to speculation itself; and its value is directly measured by the quantity of energy which it occasions—immediately in its discovery, mediately through its consequences. Life to Endymion was not preferable to death; aloof from practice, a waking error is better than a sleeping truth."*

* "Discussions," Op. cit., p. 29.

